# Turbulent Motion of Liquids in Hydraulic Resistances with a Linear Cylindrical Slide-Valve

**DOI:** 10.1155/2015/649098

**Published:** 2015-06-17

**Authors:** C. Velescu, N. C. Popa

**Affiliations:** ^1^Hydraulic Machinery Department, Mechanical Faculty, “Politehnica” University of Timisoara, Boulevard Mihai Viteazul No. 1, 300222 Timisoara, Romania; ^2^Center for Advanced and Fundamental Technical Research (CAFTR), Romanian Academy, Timisoara Branch, Boulevard Mihai Viteazul No. 24, 300223 Timisoara, Romania

## Abstract

We analyze the motion of viscous and incompressible liquids in the annular space of controllable hydraulic resistances with a cylindrical linear slide-valve. This theoretical study focuses on the turbulent and steady-state motion regimes. The hydraulic resistances mentioned above are the most frequent type of hydraulic resistances used in hydraulic actuators and automation systems. To study the liquids' motion in the controllable hydraulic resistances with a linear cylindrical slide-valve, the report proposes an original analytic method. This study can similarly be applied to any other type of hydraulic resistance. Another purpose of this study is to determine certain mathematical relationships useful to approach the theoretical functionality of hydraulic resistances with magnetic controllable fluids as incompressible fluids in the presence of a controllable magnetic field. In this report, we established general analytic equations to calculate (i) velocity and pressure distributions, (ii) average velocity, (iii) volume flow rate of the liquid, (iv) pressures difference, and (v) radial clearance.

## 1. Introduction

Constant or controllable/laminar or turbulent hydraulic resistances are components of hydraulic machinery or hydraulic elements, which comprise hydrostatic actuation or automation systems [1–3]. The hydraulic resistances are active or passive elements of the circuit and generally adjust and control the fundamental parameters of the hydraulic energy (pressure and flow rate). From a constructive view point, the controllable and turbulent hydraulic resistances are the most complex, but have the highest functional performances [1, 2, 4, 5], which is why they are particularly found in hydraulic servo system structures and proportional hydraulic devices and equipment [1–3, 5–9].

Generally, the study of hydraulic resistances considers both the constructive/geometrical [1–6, 9] and functional (the fluid motion) aspects [3–5, 7, 8, 10–12]. Thus, theoretical and experimental investigations explore the geometry of the hydraulic resistances, the optimization of their geometry, and the maximization of the control cross section [3, 6, 8, 10, 12]. A few theoretical and experimental studies approach the important problem of the cavitation phenomenon in hydraulic resistances [1, 2, 4], and other studies analyze the flow of viscous fluids through the hydraulic resistance as well as the pressure, velocity and hydrodynamic force distributions, and nature of the flow regime [3, 5, 6, 8, 10, 11].

This report theoretically analyzes the motion of viscous incompressible fluids in the annular space of controllable hydraulic resistances using a cylindrical linear slide-valve in turbulent and stationary regimes. These hydraulic resistances are the most frequent type of hydraulic resistances used in hydraulic actuators and automation systems. In the existing literature, the information refers almost strictly to the laminar motion regime [3, 4, 10, 11, 13].

The theoretical results found in the literature are generally established from the analogy of the expected Poiseuille motion [10, 11, 13, 14], and they offer the possibility of calculating the pressure difference Δ*p*, which is necessary for the flow of the flow rate *Q* through the annular space between the piston (slide-valve) and the cylinder (body), and vice versa. This annular space/radial clearance is almost always the unique solution to seal these subensembles from the hydraulic actuation, automation elements, and machineries [2, 4, 5, 8, 10, 11].

In the report, we established general analytical equations to calculate the velocity and pressure distributions, average velocity *v*_med_, volume flow rate *Q*, pressures difference Δ*p*, and radial clearance *δ*. Thus, we propose an original analytic method to study the motion of viscous incompressible fluids in controllable hydraulic resistances using a linear cylindrical slide-valve. This approach can be applied similarly to any other type of hydraulic resistance.

In fact, we consider the application of the method used in the hydrostatical or hydrodynamic lubrication field in a laminar or turbulent flow regime [12–18] to the case of the hydraulic resistance with a predominant Poiseuille motion.

First, this type of approach is motivated by the fact that the radial clearance *δ* from the hydraulic resistance is comparable to the height dimension *h* of the fluid film between the bearing surfaces [1, 2, 4, 14–18] (*δ*⇔*h*≅(10^−6^,…, 10^−5^) [m]). Additionally, in actual functional conditions, the annular space *δ* has different values in either axial or radial directions [2, 10, 11, 19, 20].

## 2. Mathematical Model

**Figure 1:**
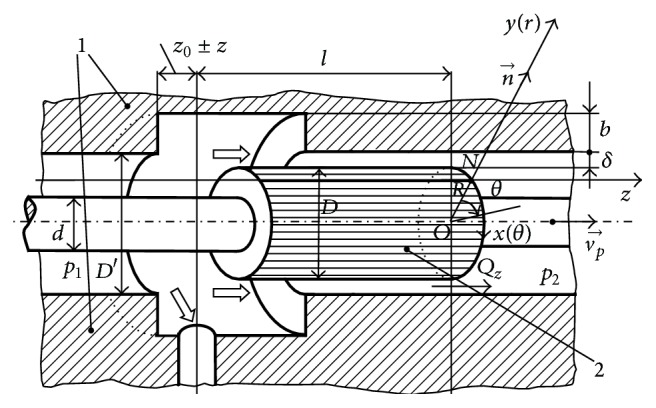
Constructive elements of the controllable turbulent hydraulic resistance with cylindrical slide-valve.

We consider the viscous incompressible liquids' motion in the annular space of the variable cross section *δ* between the cylindrical slide-valve and the body of the controllable turbulent hydraulic resistance [1, 3, 10, 11]. In transient functional regimes (closing or opening of the hydraulic resistance), the cylindrical slide-valve performs a small amplitude translation motion [1, 2, 4] in the *Oz* direction inside its body. In a stationary functional regime, the cylindrical slide-valve has a stable position versus the body of the hydraulic resistance (Figure 1).

We consider a polar cylindrical coordinate system (*z*, *r*, *θ*) or a Cartesian coordinate system (*x*, *y*, *z*) with an *Oz* direction along the length of the cylindrical slide-valve. We consider the direction *x* ≡ *R* · *θ* to be tangent to the cylindrical slide-valve. In this case, in any point on the slide-valve, the *Oy* direction is perpendicular to the cylinder's surface (Figure 1).

The mathematical model approaches the liquid's motion by considering the influence of the convective inertial forces, which depend directly on time, and the constructive-geometrical and functional particularities of these hydraulic resistances. In the mathematical results further developed, we state that the liquid motion in the annular space is a preponderant Poiseuille motion that is produced by the pressure difference Δ*p* (Δ*p* = *p*_1_ − *p*_2_; *p*_1_ > *p*_2_, where *p*_1_ is the pressure in the left side of the slide-valve).

The equations describing the motion of the viscous Newtonian incompressible fluid in the annular space from the controllable hydraulic resistances with the cylindrical slide-valve are the Navier-Stokes equation and the continuity equation. In a transient motion regime and considering the dynamical viscosity *η*≅const., these equations have the known form [13–18] as follows:

(1) $$\begin{array}{c} \frac{d\vec{V}}{d\tau }=\frac{\partial \vec{V}}{\partial \tau }+\left(\vec{V\cdot }\nabla \right)\vec{V}=\vec{f}-\frac{1}{\rho }\nabla p+\nu \Delta \vec{V};\\ \left(\nu =\frac{\eta }{\rho };\frac{\partial \vec{V}}{\partial \tau }\neq 0\right);\\ \mathrm{div}\vec{V}=\nabla \cdot \vec{V}=0. \end{array}$$

**Figure 2:**
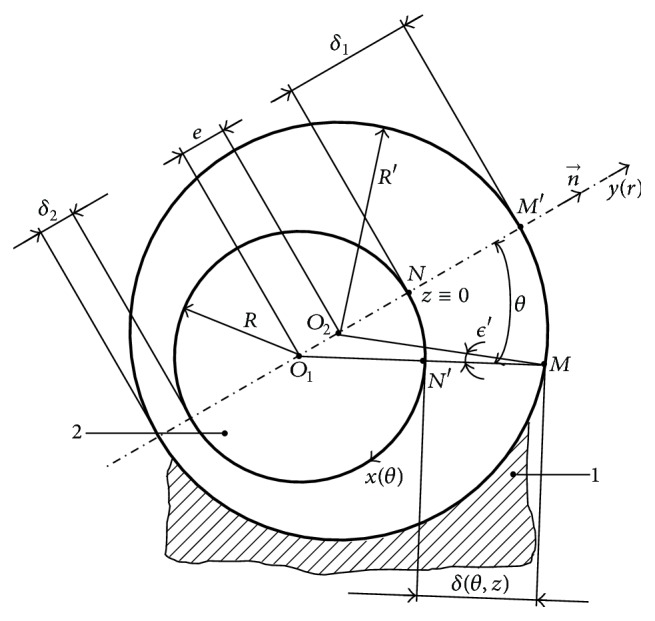
Geometrical and cinematic elements of the controllable turbulent hydraulic resistance with cylindrical slide-valve.

Because of the geometry of the hydraulic resistance with the cylindrical slide-valve (Figure 2), which is typical for movement in thin layers, the cross section of the fluid film *δ*  (*δ* ∈ [*δ*_2_ = *δ*_min_,…, *δ*_1_ = *δ*_max_]) is smaller than the other dimensions of the hydraulic resistance ((*δ*/*D*′)≅10^−3^ ≪ 1,0) (*D*′ is a characteristic dimension of the hydraulic resistance); thus, motion equations (1), written in the polar cylindrical coordinate system, will become simplified particular forms. To evaluate the magnitude order of the terms from (1), we define dimensionless variables with orders of magnitude around the unit.

Dimensionless coordinates are

(2a) $$\begin{array}{c} \bar{x}=\frac{x}{D^{\prime }};\\ \bar{y}=\frac{y}{\delta };\\ \bar{z}=\frac{z}{D^{\prime }};\\ \frac{\partial }{\partial x}=\frac{1}{R}\cdot \frac{\partial }{\partial \theta };\\ x=R\cdot \theta . \end{array}$$

Dimensionless velocities are

(2b) $$\begin{array}{c} \bar{u}=\frac{u}{U};\\ \bar{\bar{v}}=\frac{D^{\prime }}{\delta }\cdot \bar{v}=\frac{D^{\prime }}{\delta }\cdot \frac{v}{U};\\ \bar{w}=\frac{w}{U};\\ \bar{v}=\frac{v}{U} \end{array}$$

(because *u*≅*U*, *v*≅(*δ*/*D*′) · *U*, *w*≅*U*, where *U* is a characteristic velocity).

Observing the dimensionless variables (2a) and (2b) and introducing the Reynolds Poiseuille number Re_*P*_  (Re_*P*_ = *W*_med_ · *δ*/*ν* = *W*_med_ · *ρ* · *δ*/*η*) defined based on the fluid cross section *δ*, motion equations (1) (in which we neglect the mass forces $\vec{f}≅0$ because the cross section is extremely small) can be written as follows:

(3a) $$\begin{array}{c} Re_{P}\cdot \frac{\delta }{D^{\prime }}\cdot \left[\frac{D^{\prime }}{U}\cdot \frac{\partial \bar{u}}{\partial \tau }+\bar{u}\cdot \frac{1}{\bar{R}}\cdot \frac{\partial \bar{u}}{\partial \bar{\theta }}+\bar{\bar{v}}\cdot \frac{\partial \bar{u}}{\partial \bar{y}}+\bar{w}\cdot \frac{\partial \bar{u}}{\partial \bar{z}}\right]≅-\frac{\delta^{2}}{\eta \cdot \bar{R}\cdot U\cdot D^{\prime }}\cdot \frac{\partial p}{\partial \bar{\theta }}+\frac{\delta^{2}}{D^{\prime 2}}\cdot \left(\frac{1}{{\bar{R}}^{2}}\cdot \frac{\partial^{2}\bar{u}}{\partial {\bar{\theta }}^{2}}+\frac{\partial^{2}\bar{u}}{\partial {\bar{z}}^{2}}\right)+\frac{\partial^{2}\bar{u}}{\partial {\bar{y}}^{2}}; \end{array}$$

(3b) $$\begin{array}{c} Re_{P}\cdot \frac{\delta^{2}}{D^{\prime 2}}\cdot \left[\frac{D^{\prime }}{U}\cdot \frac{\partial \bar{\bar{v}}}{\partial \tau }+\bar{u}\cdot \frac{1}{\bar{R}}\cdot \frac{\partial \bar{\bar{v}}}{\partial \bar{\theta }}+\bar{\bar{v}}\cdot \frac{\partial \bar{\bar{v}}}{\partial \bar{y}}+\bar{w}\cdot \frac{\partial \bar{\bar{v}}}{\partial \bar{z}}\right]≅-\frac{\delta }{\eta \cdot U}\cdot \frac{\partial p}{\partial \bar{y}}+\frac{\delta^{3}}{D^{\prime 3}}\cdot \left(\frac{1}{{\bar{R}}^{2}}\cdot \frac{\partial^{2}\bar{\bar{v}}}{\partial {\bar{\theta }}^{2}}+\frac{\partial^{2}\bar{\bar{v}}}{\partial {\bar{z}}^{2}}\right)+\frac{\delta }{D^{\prime }}\cdot \frac{\partial^{2}\bar{\bar{v}}}{\partial {\bar{y}}^{2}}; \end{array}$$

(3c) $$\begin{array}{c} Re_{P}\cdot \frac{\delta }{D^{\prime }}\cdot \left[\frac{D^{\prime }}{U}\cdot \frac{\partial \bar{w}}{\partial \tau }+\bar{u}\cdot \frac{1}{\bar{R}}\cdot \frac{\partial \bar{w}}{\partial \bar{\theta }}+\bar{\bar{v}}\cdot \frac{\partial \bar{w}}{\partial \bar{y}}+\bar{w}\cdot \frac{\partial \bar{w}}{\partial \bar{z}}\right]≅-\frac{\delta^{2}}{\eta \cdot U\cdot D^{\prime }}\cdot \frac{\partial p}{\partial \bar{z}}+\frac{\delta^{2}}{D^{\prime 2}}\cdot \left(\frac{1}{{\bar{R}}^{2}}\cdot \frac{\partial^{2}\bar{w}}{\partial {\bar{\theta }}^{2}}+\frac{\partial^{2}\bar{w}}{\partial {\bar{z}}^{2}}\right)+\frac{\partial^{2}\bar{w}}{\partial {\bar{y}}^{2}}; \end{array}$$

  

(4) $$\begin{array}{c} \frac{1}{\bar{R}}\cdot \frac{\partial \bar{u}}{\partial \bar{\theta }}+\frac{\partial \bar{\bar{v}}}{\partial \bar{y}}+\frac{\partial \bar{w}}{\partial \bar{z}}=0. \end{array}$$

From dimensionless equation (4), we conclude that all the terms are of the same order of magnitude; thus, the continuity equation preserves the same form for the viscous-inertial motion in thin layers, and this motion is assimilated to the annular space *δ* from the hydraulic resistances [1, 2, 10]. The constructive-geometrical and functional particularities, especially for these hydraulic resistances and generally for precision hydraulic equipment, can be written as follows:

(5) $$\begin{array}{c} \frac{\delta }{D^{\prime }}\sim 10^{-3}\ll 1;\\ Re_{P}\sim 10^{3};\\ Re_{P}\cdot \frac{\delta }{D^{\prime }}⟶1.0;\\ \frac{\partial^{2}\bar{u}}{\partial {\bar{y}}^{2}}⟶1.0;\\ \frac{\partial^{2}\bar{w}}{\partial {\bar{y}}^{2}}⟶1.0;\\ U^{2}\cdot {\left(\frac{\partial \bar{u}}{\partial \bar{y}}\right)}^{2}⟶1.0;\\ U^{2}\cdot {\left(\frac{\partial \bar{w}}{\partial \bar{y}}\right)}^{2}⟶1.0. \end{array}$$

Certain terms should be neglected so that (3a), (3b), and (3c), rewritten in dimensionless variables, offer simplified equations for the motion of viscous incompressible fluids in a transient regime:

(6a) $$\begin{array}{c} \rho \cdot \left(\frac{\partial u}{\partial \tau }+\frac{u}{R}\cdot \frac{\partial u}{\partial \theta }+v\cdot \frac{\partial u}{\partial y}+w\cdot \frac{\partial u}{\partial z}\right)≅-\frac{1}{R}\cdot \frac{\partial p}{\partial \theta }+\eta \cdot \frac{\partial^{2}u}{\partial y^{2}}; \end{array}$$

(6b) $$\begin{array}{c} \frac{\partial p}{\partial y}≅0; \end{array}$$

(6c) $$\begin{array}{c} \rho \cdot \left(\frac{\partial w}{\partial \tau }+\frac{u}{R}\cdot \frac{\partial w}{\partial \theta }+v\cdot \frac{\partial w}{\partial y}+w\cdot \frac{\partial w}{\partial z}\right)≅-\frac{\partial p}{\partial z}+\eta \cdot \frac{\partial^{2}w}{\partial y^{2}}; \end{array}$$

(6d) $$\begin{array}{c} \frac{1}{R}\cdot \frac{\partial u}{\partial \theta }+\frac{\partial v}{\partial y}+\frac{\partial w}{\partial z}=0. \end{array}$$

Equations (6a), (6b), (6c), and (6d) are valid for motions in thin layers for hydraulic resistances with a cylindrical slide-valve and consider all inertial forces. From (6b), we can observe that the pressure *p* is constant over the thickness of the liquid layer *δ*.

## 3. Integration of the Motion Equations

To calculate the velocity and pressure distributions in the *z* direction (Figure 1), we must analyze the fluid motion in the annular space *δ* between the slide-valve and the body of the hydraulic resistance. We will use (6a), (6b), (6c), and (6d) which will be integrated along the length of the thickness of the fluid layer. We will perform an integration of the equations with partial derivatives (6a), (6b), (6c), and (6d) using the approximate method proposed by S. M. Targ and N. A. Slezkin and found in literature [13, 14, 17, 18]. We consider that the fluid motion in the annular space between the slide-valve and the body (Figure 1) is described by the velocity parabolic profiles *u*(*y*), *v*(*y*), and *w*(*y*), which qualitatively maintain the same appeal as the noninertial case:

(7a) $$\begin{array}{c} u\left(y\right)≅-\frac{1}{2\cdot \eta }\cdot \frac{1}{R}\cdot \frac{\partial p}{\partial \theta }\cdot y\cdot \left(\delta -y\right); \end{array}$$

(7b) $$\begin{array}{c} v\left(y\right)≅0; \end{array}$$

(7c) $$\begin{array}{c} w\left(y\right)≅-\frac{1}{2\cdot \eta }\cdot \frac{\partial p}{\partial z}\cdot y\cdot \left(\delta -y\right)+v_{p}\cdot \left(1-\frac{y}{\delta }\right), \end{array}$$

where the slide-valve is considered to have a small amplitude translational motion in the horizontal direction (closing/opening), along the *z*-axis, with a linear velocity *v*_*p*_.

The velocity distributions (7a), (7b), and (7c) describe a laminar motion regime [11, 12, 15, 16, 18]. In the turbulent motion regime case, the velocity distributions can be described as follows [12, 15–17]:

(8a) $$\begin{array}{c} u\left(y\right)≅-\frac{1}{\eta }\cdot \frac{1}{k_{x}^{o}}\cdot \frac{1}{R}\cdot \frac{\partial p}{\partial \theta }\cdot y\cdot \left(\delta -y\right); \end{array}$$

(8b) $$\begin{array}{c} v\left(y\right)≅0; \end{array}$$

(8c) $$\begin{array}{c} w\left(y\right)≅-\frac{1}{\eta }\cdot \frac{1}{k_{z}^{o}}\cdot \frac{\partial p}{\partial z}\cdot y\cdot \left(\delta -y\right)+v_{p}\cdot \left(1-\frac{y}{\delta }\right). \end{array}$$

For the velocity distributions, the boundary conditions corresponding to the motion described by (7a), (7b), (7c), (8a), (8b), and (8c) can be given as follows:

(i) for *y* = 0:
(9a) $$\begin{array}{c} u≅0;\\ v≅0;\\ w≅v_{p}; \end{array}$$
(ii) for *y* = *δ*[*x*(*θ*); *z*]:
(9b) $$\begin{array}{c} u≅0;\\ v≅0;\\ w≅0. \end{array}$$

We can consider that the resultant motion between the slide-valve and the body is a predominant Poiseuille motion, and we can introduce the average velocities for the fluid as *U*_med_, *V*_med_, and *W*_med_ and the fluid unitary flow rates as *Q*_*θ*_, *Q*_*y*_, and *Q*_*z*_, which cross the space *δ* of unitary width in *θ*, *y*(*r*), and *z* directions, respectively:

(10a) $$\begin{array}{c} U_{med}=\frac{1}{\delta }\cdot \overset{\delta }{\underset{0}{\int }}u\left(y\right)dy;\\ V_{med}=\frac{1}{\delta }\cdot \overset{\delta }{\underset{0}{\int }}v\left(y\right)dy;\\ W_{med}=\frac{1}{\delta }\cdot \overset{\delta }{\underset{0}{\int }}w\left(y\right)dy; \end{array}$$

(10b) $$\begin{array}{c} Q_{\theta }≅U_{med}\cdot \delta ≅-\frac{\delta^{3}}{12\cdot \eta }\cdot \frac{1}{R}\cdot \frac{\partial p}{\partial \theta };\\ Q_{y}\equiv Q_{r}≅0;\\ Q_{z}≅W_{med}\cdot \delta ≅-\frac{\delta^{3}}{12\cdot \eta }\cdot \frac{\partial p}{\partial z}+v_{p}\cdot \frac{\delta }{2}. \end{array}$$

Equation (10b) describes a laminar motion regime [11, 12, 15, 16, 18]. For a turbulent motion regime, (10b) becomes [12, 15–17]

(11) $$\begin{array}{c} Q_{\theta }≅U_{med}\cdot \delta ≅-\frac{1}{\eta }\cdot \frac{\delta^{3}}{k_{x}}\cdot \frac{1}{R}\cdot \frac{\partial p}{\partial \theta };\\ Q_{y}\equiv Q_{r}≅0;\\ Q_{z}≅W_{med}\cdot \delta ≅-\frac{1}{\eta }\cdot \frac{\delta^{3}}{k_{z}}\cdot \frac{\partial p}{\partial z}+v_{p}\cdot \frac{\delta }{2}. \end{array}$$

In (8a), (8b), and (8c) we can denote the turbulent flow “local” parameters with *k*_*x*_^*o*^ and *k*_*z*_^*o*^, which can be formally defined by the following relationships [15–17]:

(12a) $$\begin{array}{c} k_{x}^{o}≅\frac{-1/\eta \cdot 1/R\cdot \partial p/\partial \theta \cdot y\cdot \left(\delta -y\right)\cdot dy}{d\left(-y^{3}/\left(\eta \cdot k_{x}\right)\cdot 1/R\cdot \partial p/\partial \theta \right)}; \end{array}$$

(12b) $$\begin{array}{c} k_{z}^{o}≅\frac{-1/\eta \cdot \partial p/\partial z\cdot y\cdot \left(\delta -y\right)\cdot dy}{d\left(-y^{3}/\left(\eta \cdot k_{z}\right)\cdot \partial p/\partial z+v_{p}\cdot y/2\right)-v_{p}\cdot \left(1-y/\delta \right)dy}. \end{array}$$

In the laminar motion regime, parameters *k*_*x*_^*o*^ and *k*_*z*_^*o*^ have a value of *k*_*x*_^*o*^ = *k*_*z*_^*o*^ = 2. In the absence of other bibliographical recommendations, in a turbulent motion regime, we can assume the same values of *k*_*x*_^*o*^≅*k*_*z*_^*o*^≅2 [15–17].

In (11), (12a), and (12b), we can denote the turbulent flow “global” parameters by *k*_*x*_ and *k*_*z*_ or more simply the turbulent flow parameters [12–17]. These parameters can be determined as follows [15–17]:

(13a) $$\begin{array}{c} k_{x}=12+0.53\cdot {\left[{\left(0.193479573-6.06787\cdot 10^{-7}\cdot Re_{P}\right)}^{2}\cdot \left(Re_{P}-2000\right)\right]}^{0.725}; \end{array}$$

(13b) $$\begin{array}{c} k_{z}=12+0.296\cdot {\left[{\left(0.387564278-8.99509\cdot 10^{-7}\cdot {Re}_{P}\right)}^{2}\cdot \left({Re}_{P}-2000\right)\right]}^{0.65}, \end{array}$$

where Re_*P*_ ∈ [2000,…, 10^5^).

Or, more generally, the following relations are known:

(14a) $$\begin{array}{c} k_{x}≅12+0.53\cdot {\left[{\left(0.194693813-6.067946\cdot 10^{-7}\cdot Re_{P}-6.06787\cdot 10^{-7}\cdot Re_{Pcr}\right)}^{2}\cdot \left(Re_{P}-Re_{Pcr}\right)\right]}^{0.725}; \end{array}$$

(14b) $$\begin{array}{c} k_{z}≅12+0.296\cdot {\left[{\left(0.389363298-8.995085\cdot 10^{-7}\cdot Re_{P}-8.99509\cdot 10^{-7}\cdot Re_{Pcr}\right)}^{2}\cdot \left(Re_{P}-Re_{Pcr}\right)\right]}^{0.65}, \end{array}$$

where Re_Pcr_ ∈ [1700,…, 2000(2200)] and Re_*P*_ ∈ [1700,…, 10^5^).

In the case of the laminar motion regime, for Re_*P*_ = 2000, the flow parameters *k*_*x*_ and *k*_*z*_ have a value of *k*_*x*_ = *k*_*z*_ = 12.

In the literature [9, 12, 14, 21–23] there are also other calculus relations for *k*_*x*_ and *k*_*z*_ but we demonstrated (theoretically and experimentally) [15–17] that these calculus relations lead to theoretical values very different from those experimentally obtained.

Furthermore, we can consider that the velocity *v*_*p*_≅0 and that predominant motion of the liquid is in the *z* direction (Figure 1). In the literature, there are several mathematical relationships used to calculate the fluid flow rate *Q*_*z*_ in direction *z* [1, 2, 4, 5, 8]. For example, we can state the relationship [2] as follows:

(15) $$\begin{array}{c} Q_{z}≅\frac{\pi \cdot D_{med}\cdot \delta_{med}^{3}}{12\cdot \rho \cdot \nu \cdot l}\cdot \left(1+\frac{3}{2}\cdot \frac{e^{2}}{\delta_{med}^{2}}\right)\cdot \Delta p, \end{array}$$

where *D*_med_ = (*D*′ + *D*)/2, *δ*_med_ = (*δ*_max_ + *δ*_min_)/2 = (*δ*_1_ + *δ*_2_)/2, Δ*p* = *p*_1_ − *p*_2_, *p*_1_ > *p*_2_, and *ν* = *η*/*ρ*.

If the cylindrical linear slide-valve and the body are disposed concentrically, then the eccentricity *e* = 0 and (15) becomes

(16) $$\begin{array}{c} Q_{z}≅\frac{\pi \cdot D_{med}\cdot \delta_{med}^{3}}{12\cdot \rho \cdot \nu \cdot l}\cdot \Delta p. \end{array}$$

It should be noted that by using the theoretical and experimental results from literature [13, 14, 22, 23] we acknowledge that the deviations from the parabolic profiles of the velocities are greater when the Reynolds Poiseuille (Re_*P*_) number is larger than Re_Pcr_  (Re_Pcr_≅2000).

By substituting (8a), (8b), and (8c) with *v*_*p*_≅0 and integrating (6a), (6b), (6c), and (6d) term by term, we obtain the final expressions as follows:

(17a) $$\begin{array}{c} \rho \cdot \frac{\partial }{\partial \tau }\left(U_{med}\cdot \delta \right)+\rho \cdot \frac{1}{R}\cdot \frac{\partial }{\partial \theta }\left(\alpha_{0}\cdot U_{med}^{2}\cdot \delta \right)+\rho \cdot \frac{\partial }{\partial z}\left(\alpha_{1}\cdot U_{med}\cdot W_{med}\cdot \delta \right)+\frac{1}{R}\cdot \frac{\partial p}{\partial \theta }\cdot \delta +\frac{2\cdot k_{x}}{k_{x}^{o}\cdot \delta }\cdot U_{med}\cdot \eta ≅0; \end{array}$$

(17b) $$\begin{array}{c} \rho \cdot \frac{\partial }{\partial \tau }\left(W_{med}\cdot \delta \right)+\rho \cdot \frac{1}{R}\cdot \frac{\partial }{\partial \theta }\left(\alpha_{1}\cdot U_{med}\cdot W_{med}\cdot \delta \right)+\rho \cdot \frac{\partial }{\partial z}\left(\alpha_{2}\cdot W_{med}^{2}\cdot \delta \right)+\frac{\partial p}{\partial z}\cdot \delta +\frac{2\cdot k_{z}}{k_{z}^{o}\cdot \delta }\cdot W_{med}\cdot \eta ≅0; \end{array}$$

(17c) $$\begin{array}{c} \frac{1}{R}\cdot \frac{\partial }{\partial \theta }\left(U_{med}\cdot \delta \right)+\frac{\partial }{\partial z}\left(W_{med}\cdot \delta \right)≅0, \end{array}$$

where we used the following notations [15–17]:

(18) $$\begin{array}{c} \alpha_{0}=\frac{k_{x}^{2}}{30\cdot k_{x}^{o2}};\\ \alpha_{1}=\frac{k_{x}\cdot k_{z}}{30\cdot k_{x}^{o}\cdot k_{z}^{o}};\\ \alpha_{2}=\frac{k_{z}^{2}}{30\cdot k_{z}^{o2}}. \end{array}$$

The equations with partial derivatives (17a), (17b), and (17c) along with the Reynolds equation [15, 16] if necessary can solve the problem of the motion of incompressible viscous fluids in hydraulic resistances with a linear cylindrical slide-valve under a turbulent regime while considering all the inertial forces (i.e., determining the hydrodynamic variables *U*_med_, *W*_med_, and *p*). For the turbulent and steady-state motion regimes, the terms in (17a), (17b), and (17c) that contain the partial derivative with respect to the time *τ* are zero.

If the parameters in (18) are *α*_0_ = *α*_1_ = *α*_2_ = 0 and we consequently completely neglect the inertial effects, the system of equations in (17a), (17b), and (17c) will be reduced to a noninertial system of equations, which will result in the following equations:

(19a) $$\begin{array}{c} \frac{1}{R}\cdot \frac{\partial p}{\partial \theta }\cdot \delta +\frac{2\cdot k_{x}}{k_{x}^{o}\cdot \delta }\cdot U_{med}\cdot \eta ≅0; \end{array}$$

(19b) $$\begin{array}{c} \frac{\partial p}{\partial z}\cdot \delta +\frac{2\cdot k_{z}}{k_{z}^{o}\cdot \delta }\cdot W_{med}\cdot \eta ≅0; \end{array}$$

$$\begin{array}{cc} \left(17c\right) & \frac{1}{R}\cdot \frac{\partial }{\partial \theta }\left(U_{med}\cdot \delta \right)+\frac{\partial }{\partial z}\left(W_{med}\cdot \delta \right)≅0. \end{array}$$

Using the global hydrodynamic parameters *U*_med_, *W*_med_, and *p*, we can calculate the pressure and velocity distributions and the volume unitary fluid flow rate *Q*_*z*_ from the hydraulic resistance. Based on these calculations, we can determine the fundamental functional parameters of the hydraulic resistances with a linear cylindrical slide-valve [1, 2, 4, 5, 8] in a turbulent motion regime.

The hypothesis admitted above concerning the parabolic velocity profiles [15–17] is not rigorously true [13, 14]. Consequently, we must consider that the “inertial” parameters in (18) (*α*_0_, *α*_1_, and *α*_2_) are functions of variables *x* and *z*. Hence, they were written in parenthesis in (17a), (17b), and (17c). Furthermore, by observing (12a), (12b), (13a), (13b), and (14a), (14b), we can note that the “inertial” parameters in (18) are much more complex functions. These parameters depend not only on the *x* and *z* variables but also on the “local” and “global” parameters of the turbulent flow in (12a), (12b), (13a), (13b), and (14a), (14b), which are functions of the “local” and “global” Reynolds Poiseuille number (Re_*P*_^*o*^ and Re_*P*_) [2, 15–17].

Additionally, for the coefficients *k*_*x*_, *k*_*z*_, *k*_*x*_^*o*^, and *k*_*z*_^*o*^, if we consider the values corresponding to the laminar motion regime, that is, *k*_*x*_ = *k*_*z*_ = 12 and *k*_*x*_^*o*^ = *k*_*z*_^*o*^ = 2, then we can determine that *α*_0_ = *α*_1_ = *α*_2_ = 6/5, which indicates that we can determine the values of the inertial coefficients from the laminar motion regime, *α*_0_ = 6/5 [15–18].

This result indicates that the differences between the mathematical model established for the laminar motion regime and the mathematical model established for the turbulent motion regime are specified exactly by these inertial coefficients [15–17].

The above results indicate a much higher complexity of the viscous fluids' motion in hydraulic resistances with a cylindrical linear slide-valve under a turbulent regime compared to the laminar motion regime [2, 4, 11].

## 4. Pressure Distribution in a Space along *z* Direction

The differential equation for the pressure distributions versus the *z* direction will be established from the equations of the viscous fluids' motion in the hydraulic resistances with a linear cylindrical slide-valve under a turbulent regime. These equations are established in Section 3.

Thus, by examining the equations with partial derivatives (17a), (17b), and (17c) (which correspond to a steady-state and turbulent motion regime) and assuming that the functioning regime is stationary (the body and the cylindrical slide-valve are fixed) and the predominant motion of the fluid is in the direction of the *z*-axis, Δ*p* = *p*_1_ − *p*_2_, *p*_1_ > *p*_2_ (Figure 1), then the average velocity *U*_med_≅0 in (17a), (17b), and (17c). Under these conditions, differential equation (17b) (which corresponds to a pure motion in the length of *z* direction) can be written in the particular form as follows:

(20) $$\begin{array}{c} \rho \cdot \frac{\partial }{\partial z}\left(\alpha_{2}\cdot W_{med}^{2}\cdot \delta \right)+\frac{\partial p}{\partial z}\cdot \delta +\frac{2\cdot k_{z}}{k_{z}^{o}\cdot \delta }\cdot W_{med}\cdot \eta ≅0. \end{array}$$

Considering (11), (20) becomes

(21) $$\begin{array}{c} \rho \cdot \frac{\partial }{\partial z}\left(\alpha_{2}\cdot \frac{Q_{z}^{2}}{\delta }\right)+\frac{\partial p}{\partial z}\cdot \delta +\frac{k_{z}\cdot 2}{k_{z}^{o}\cdot \delta^{2}}\cdot Q_{z}\cdot \eta ≅0, \end{array}$$

or equivalently

(22) $$\begin{array}{c} \rho \cdot \frac{d}{dz}\left(\alpha_{2}\cdot \frac{Q_{z}^{2}}{\delta }\right)+\frac{dp}{dz}\cdot \delta +\frac{2\cdot k_{z}}{k_{z}^{o}\cdot \delta^{2}}\cdot Q_{z}\cdot \eta ≅0. \end{array}$$

We can derive (22) with respect to the *z* variable while considering the constant and variable parameters from the mathematical relationship and that the radial dimension *δ* varies along the *z* direction [2, 17, 18].

In the following section, we assume (the most frequent situation in the field of components for hydrostatical systems with actuation and automation) that the slide-valve diameter, *D*, increases in the direction of the liquid flow when the radial dimension *δ* decreases from a value *δ* = *δ* ^*∗*^ for *z* = *l* = 0 to a value *δ* = *δ* ^*∗∗*^ for the length *l* of the slide-valve (Figure 1). It should be noted that this constructive-functioning situation is to our advantage because a cylindrical slide-valve with a diameter *D* that increases in the direction of the liquid flow will center itself and thus benefit the pressure distribution, unlike the reverse situation where it is possible that the slide-valve adheres to the body [2, 4–6, 10]. Additionally, it can be noted that the variation in the slide-valve diameter *D* versus the length *l* is slight, that is, on the order of microns [1, 2, 4].

Therefore, the final expression can be written as follows:

(23) $$\begin{array}{c} \frac{dp}{dz}≅\rho \cdot \alpha_{2}\cdot Q_{z}^{2}\cdot \frac{1}{\delta^{3}}\cdot \frac{d\delta }{dz}-\frac{2\cdot k_{z}}{k_{z}^{o}\cdot \delta^{3}}\cdot Q_{z}\cdot \eta . \end{array}$$

In (23), the volume flow rate *Q*_*z*_ can be considered to be a constant, and if we know the geometrical elements, the working fluid, and the value of the pressure difference Δ*p* = *p*_1_ − *p*_2_ (Δ*p* = const. for an established geometry) this constant can be determined using (15) and (16). Equation (23) can be written in the equivalent form as follows:

(24) $$\begin{array}{c} dp≅\rho \cdot \alpha_{2}\cdot Q_{z}^{2}\cdot \frac{1}{\delta^{3}}\cdot d\delta -\frac{2\cdot k_{z}}{k_{z}^{o}\cdot \delta^{3}}\cdot Q_{z}\cdot \eta \cdot dz. \end{array}$$

We can now integrate (24) by considering the constructive-functional boundary conditions already determined for *z* = 0, *p* = *p*_1_, and *δ* = *δ* ^*∗*^ and *z* = *l*, *p* = *p*_2_, and *δ* = *δ* ^*∗∗*^ (Δ*p* = *p*_1_ − *p*_2_; *p*_1_ > *p*_2_; *δ* ^*∗*^ > *δ* ^*∗∗*^).

First, we can observe the relationship between the geometrical elements (Figure 1):

(25) $$\begin{array}{c} \delta ≅\delta^{*}-\frac{\delta^{*}-\delta^{**}}{l}\cdot z. \end{array}$$

Using (25), (24) becomes

(26) $$\begin{array}{c} dp≅\left(\rho \cdot \alpha_{2}\cdot Q_{z}^{2}+\frac{2\cdot k_{z}}{k_{z}^{o}}\cdot \frac{l}{\delta^{*}-\delta^{**}}\cdot Q_{z}\cdot \eta \right)\cdot \frac{d\delta }{\delta^{3}}. \end{array}$$

By integrating (26), we can obtain

(27) $$\begin{array}{c} p\left(z\right)\equiv p\left(\delta \right)≅p_{1}+\left(\rho \cdot \alpha_{2}\cdot Q_{z}^{2}+\frac{2\cdot k_{z}}{k_{z}^{o}}\cdot \frac{l}{\delta^{*}-\delta^{**}}\cdot Q_{z}\cdot \eta \right)\cdot \overset{\delta }{\underset{\delta^{*}}{\int }}\frac{d\delta }{\delta^{3}}, \end{array}$$

or, by integrating the right side of the anterior relation, we can obtain

(28) $$\begin{array}{c} p\left(z\right)\equiv p\left(\delta \right)≅p_{1}-\left(\rho \cdot \alpha_{2}\cdot Q_{z}^{2}+\frac{2\cdot k_{z}}{k_{z}^{o}}\cdot \frac{l}{\delta^{*}-\delta^{**}}\cdot Q_{z}\cdot \eta \right)\cdot \frac{1}{2}\cdot \left(\frac{\delta^{*2}-\delta^{2}}{\delta^{*2}\cdot \delta^{2}}\right). \end{array}$$

For *z* = *l*, *p*(*z*) ≡ *p* = *p*_2_, and *δ* = *δ* ^*∗∗*^, we can obtain from the last relation

(29) $$\begin{array}{c} p_{2}≅p_{1}-\left(\rho \cdot \alpha_{2}\cdot Q_{z}^{2}+\frac{2\cdot k_{z}}{k_{z}^{o}}\cdot \frac{l}{\delta^{*}-\delta^{**}}\cdot Q_{z}\cdot \eta \right)\cdot \frac{1}{2}\cdot \left(\frac{\delta^{*2}-\delta^{**2}}{\delta^{*2}\cdot \delta^{**2}}\right). \end{array}$$

Furthermore, the pressure difference Δ*p* can be given as follows:

(30) $$\begin{array}{c} \Delta p=p_{1}-p_{2}≅\left(\rho \cdot \alpha_{2}\cdot Q_{z}^{2}+\frac{2\cdot k_{z}}{k_{z}^{o}}\cdot \frac{l}{\delta^{*}-\delta^{**}}\cdot Q_{z}\cdot \eta \right)\cdot \frac{1}{2}\cdot \left(\frac{\delta^{*2}-\delta^{**2}}{\delta^{*2}\cdot \delta^{**2}}\right). \end{array}$$

Equation (30) offers a functional relationship between the necessary pressure difference Δ*p* and the unitary volume flow rate *Q*_*z*_, which flows in a turbulent regime through the ring-shaped space *δ*, *δ* ∈ [*δ* ^*∗∗*^ ≡ *δ*_min_,…, *δ* ^*∗*^ ≡ *δ*_max_], when the cylindrical linear slide-valve is concentrically placed in the hydraulic resistance body.

In [11] we find a similar equation (to equation (30)), but for the laminar regime.

For an established hydraulic resistance and a function of the served hydrostatical actuating and automation systems, we can determine all the parameters except for the pressure difference Δ*p* and the unitary volume flow rate *Q*_*z*_.

The mathematical relationships from the literature ((15) and (16)) were established using a different approach and correspond to the hydraulic resistances with a cylindrical linear slide-valve under a laminar regime with a perfectly cylindrical slide-valve disposed (in the resistance body) eccentrically (15) or concentrically (16).

By considering only the simple case when the cylindrical slide-valve is placed concentrically, (16) (corresponding to the unitary flow rate *Q*_*z*_) and (30) can be used to completely solve the fluid flow through the ring-shaped space *δ* and determine the unknown quantities Δ*p* and *Q*_*z*_.

At the limit, (16) can also be used for a turbulent flow regime; in the case of a circular linear slide-valve, the diameter *D* (Figure 1) “moderately” increases in the sense of the fluid flow *z*. The slide-valve length *l* cannot be extremely large, as will be demonstrated further.

## 5. Numerical Results

Equation (16), which was rewritten for the unitary volume flow rate *Q*_*z*_ for the turbulent flow regime and for a cylindrical slide-valve with diameter *D*, which increases “moderately” in the sense of the liquid flow *z*, can be written in the following form [2, 14, 15]:

(31) $$\begin{array}{c} Q_{z}≅\frac{\delta_{med}^{3}\cdot \Delta p}{k_{z}\cdot \rho \cdot \nu \cdot l}=\frac{\delta_{med}^{3}\cdot \Delta p}{k_{z}\cdot \eta \cdot l},\\ \left(\nu =\frac{\eta }{\rho }\right), \end{array}$$

where *δ*_med_≅(*δ*_min_ + *δ*_max_)/2 = (*δ* ^*∗∗*^ + *δ* ^*∗*^)/2, and the flow coefficient in the turbulent regime *k*_*z*_ can be determined using (13b) or, more generally, (14b).

By replacing Δ*p* from (31) in (30), we can obtain an algebraic equation of second degree in *Q*_*z*_ as follows:

(32) $$\begin{array}{c} \left(\rho \cdot \alpha_{2}\cdot Q_{z}^{2}+\frac{2\cdot k_{z}}{k_{z}^{o}}\cdot \frac{l}{\delta^{*}-\delta^{**}}\cdot Q_{z}\cdot \eta \right)\cdot \frac{\delta^{*2}-\delta^{**2}}{2\cdot \delta^{*2}\cdot \delta^{**2}}-\frac{8\cdot Q_{z}\cdot k_{z}\cdot \eta \cdot l}{{\left(\delta^{*}+\delta^{**}\right)}^{3}}≅0. \end{array}$$

From this relation, we can obtain

(33) $$\begin{array}{c} Q_{z}≅\frac{2\cdot k_{z}\cdot \eta \cdot l}{\rho \cdot \alpha_{2}}\cdot \left[\frac{8\cdot \delta^{*2}\cdot \delta^{**2}}{\left(\delta^{*2}-\delta^{**2}\right)\cdot {\left(\delta^{*}+\delta^{**}\right)}^{3}}-\frac{1}{k_{z}^{o}\cdot \left(\delta^{*}-\delta^{**}\right)}\right]. \end{array}$$

Equations (31) and (33) allow the hydrodynamic unknowns Δ*p* and *Q*_*z*_ to be calculated for any hydraulic resistance with a linear cylindrical sliding valve under a turbulent regime with the sliding valve geometry as described above. The problem is similar for any other geometry of the sliding valve for hydraulic resistance.

In many practical situations [1–4, 6], the function of the destination, the functional technical requests of the automation and acting hydrostatical systems, and the pressure difference Δ*p* are imposed; thus, (30) can be used to determine an exact calculation of the unitary volume flow rate *Q*_*z*_.

The other parameters from (30), (31), and (33) are known and have typical values as mentioned in the literature ([1, 2, 4, 6, 8, 13], and others).

Thus, for the numerical calculations we considered the following typical values: (i) *η*≅5 · 10^−2^ [(N/m^2^) · s ≡ Pa · s] (mineral oil H30, standard STAS 9691-94) [2–4]; (ii) *ρ*≅905 [kg/m^3^] [2–4]; (iii) *l*≅0.020 [m] [1, 2, 4]; (iv) *δ* ^*∗*^≅(0.001,…, 0.04) [mm] [1, 2, 4]; (v) *δ* ^*∗∗*^≅(0.0,…, *δ* ^*∗*^) [mm] [1, 2, 4]; (vi) Re_*P*_≅1500  [−] [1, 2]; (vii) Re_Pcr_≅1000 [−] [1, 2]; (viii) *k*_*z*_^*o*^≅2.0 [2, 9]; (ix) *α*_2_≅2.379 [2, 15] and

(34) $$\begin{array}{c} k_{z}≅12+0.296\cdot {\left[{\left(0.389363298-8.995085\cdot 10^{-7}\cdot {Re}_{P}-8.99509\cdot 10^{-7}\cdot {Re}_{Pcr}\right)}^{2}\cdot \left({Re}_{P}-{Re}_{Pcr}\right)\right]}^{0.65}≅16.89568578. \end{array}$$

**Figure 3:**
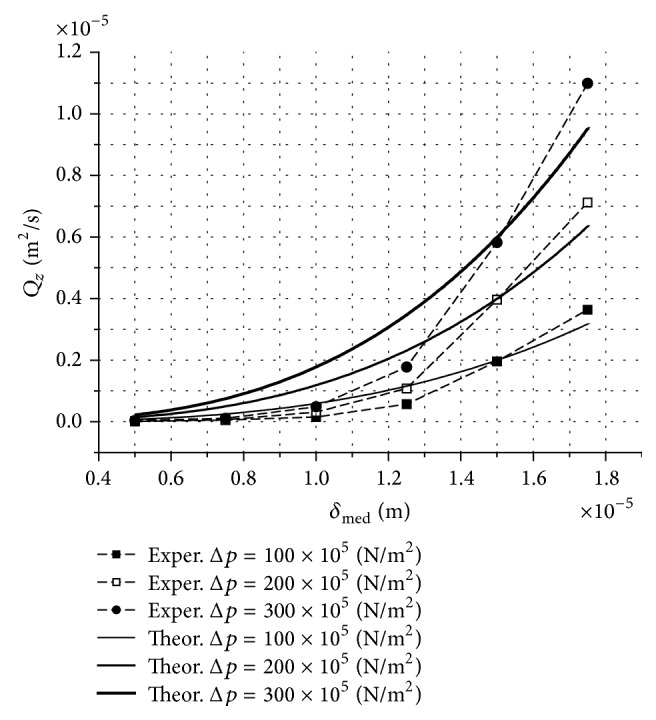
Theoretical and experimental values of *Q*_*z*_ versus *δ*_med_, with Δ*p* as the parameter (experimental values from [3]; see Table 1).

A part of the theoretical results obtained in this study is shown in Figure 3.

## 6. Comparison between the Theoretical and Experimental Results

We compared the theoretical results obtained in this report with the experimental results found in the literature. In [3], the author performed an impressive volume of theoretical and experimental studies concerning the hydrologistors as new hydraulic systems and elements. For the experimental studies, including the measurements of the liquid volume flow rates, the author used a very complex experimental set-up, built using tens of transducers and hydraulic or electronic designs. Between these, a turbine flow rate transducer (TURBOQUANT) has 1% accuracy. For this transducer, the correlation between the flow rate and the number of rotations per minute (of the turbine) was established through calibration, using a standard having its accuracy better than 0.4%. The values of the measured volume flow rates resulted as the arithmetical average of three different measurements for the same pressure difference at the same radial clearance *δ* between the linear cylindrical slide-valve and the corp. In this complex experimental set-up, considering all the efforts made for a good accuracy of the measurements, we estimate the total error of these measurements in ±3%. In Table 1, we centralized a small part of the experimental results published in [3]. We used these experimental results for a comparative study. This comparative analysis is provided in Figure 3.

## 7. Discussions and Conclusions

- The report approaches the theoretical study of the movement of incompressible viscous fluids in the inner space of adjustable hydraulic resistors with a linear cylindrical slide-valve under steady-state and turbulent regimes. We established general analytical relationships to calculate the distributions of the velocities and pressures, average velocities, flow rate, pressure differences, and so forth.
- The established analytical relationships indicate that in a turbulent regime the values of the main hydrodynamics parameters (*Q*_*z*_, Δ*p*, *W*_med_, etc.) are generally greater than those in a laminar regime.
- If we consider the proper values of the laminar movement regime (*k*_*z*_ = 12, *k*_*z*_^*o*^ = 2, and *α*_2_ = 6/5 = 1.2), the analytical relationships established in this report correspond to this movement regime.
- For a rigorous hydraulic calculation, the values of the parameters *k*_*z*_^*o*^, *k*_*z*_, and *α*_2_ and thus the values of the Reynolds Poiseuille number Re_*P*_ must be calculated for each value of the inner dimension *δ* and each value of the unitary volume flow rate *Q*_*z*_.
- Theoretical studies and experimental measurements [15–17] proved that, for the parameters *k*_*z*_ and *k*_*x*_ (*k*_*θ*_), the calculus relations found in literature [9, 14, 21–23] lead to theoretical values at least ten times higher than those experimentally obtained.
- The relationships established in this report can also be used (with an acceptable error) in the case of a moderate eccentricity because, in the case of a small circumferential unitary extinction, the influence of the eccentricity is small.
- The obtained numerical results indicate that the unitary flow rate passing through the inner space *δ* (between the slide-valve and the body) increases when the pressure difference Δ*p* increases and implicitly increases when the value of *δ*_med_ increases.
- The comparative analysis of the theoretical end experimental results shows a good correlation between the experiment and the mathematical model proposed in this report, especially for moderate values of *δ* and Δ*p*.
- The theoretical and experimental results presented above show that the unitary volume flow rate *Q*_*z*_ can be neglected for *δ* < 5 · 10^−6^ [m] (*δ* < 5 [*μ*m]). In fact, normal operation of the hydraulic resistance (avoiding blocked movement) always imposes the existence of a minimum clearance (*δ* ~ 10^−6^ [m]) and implies a unitary volume flow rate *Q*_*z*_ > 0 that is as small as possible.

## Figures and Tables

**Table 1:** Experimental data [3].

*Q*_*z*_ [m^2^/s] for Δ*p* = 100 · 10^5^ [N/m^2^]	3.64 · 10^−6^	1.96 · 10^−6^	5.68 · 10^−7^	1.57 · 10^−7^	5.12 · 10^−8^	1.09 · 10^−8^
*Q*_*z*_ [m^2^/s] for Δ*p* = 200 · 10^5^ [N/m^2^]	7.12 · 10^−6^	3.96 · 10^−6^	1.08 · 10^−6^	3.12 · 10^−7^	6.10 · 10^−8^	1.92 · 10^−8^
*Q*_*z*_ [m^2^/s] for Δ*p* = 300 · 10^5^ [N/m^2^]	1.099 · 10^−5^	5.82 · 10^−6^	1.78 · 10^−6^	4.86 · 10^−7^	1.075 · 10^−7^	3.86 · 10^−8^
*δ*_med_ [m]	17.5 · 10^−6^	15.0 · 10^−6^	12.5 · 10^−6^	10.0 · 10^−6^	7.5 · 10^−6^	5.0 · 10^−6^

## Nomenclature

*D*: The exterior diameter of the linear cylindrical slide-valve [m]
*D* ^′^: The interior diameter of the exterior carcass of the hydraulic resistance [m]
*e*: The eccentricity [m]
*l*: The length of the linear cylindrical slide-valve [m]
*Q*_*z*_: The unitary volume flow rate that flows through the space *δ* [m^2^/s]
Re_*P*_: The Reynolds Poiseuille number [−]
Re_Pcr_: The Reynolds Poiseuille critical number [−]
*ν*: The kinematic viscosity of the liquid [m^2^/s]
Δ*p*: The pressure difference on the linear cylindrical slide-valve [N/m^2^]
*δ*: The annular space between the slide-valve and the exterior carcass of the hydraulic resistance [m]
*δ* ^*∗*^: The maximum value of *δ* [m]
*δ* ^*∗∗*^: The minimum value of *δ* [m]
*η*: The dynamic viscosity of the liquid [N · s/m^2^]
*ρ*: The density of the liquid [kg/m^3^]
*τ*: The time [s].
